# Pyrite-Type CoS_2_ Nanoparticles Supported on Nitrogen-Doped Graphene for Enhanced Water Splitting

**DOI:** 10.3389/fchem.2018.00569

**Published:** 2018-11-21

**Authors:** Wei Zhang, Xiaoya Ma, Cheng Zhong, Tianyi Ma, Yida Deng, Wenbin Hu, Xiaopeng Han

**Affiliations:** ^1^Tianjin Key Laboratory of Composite and Functional Materials, School of Materials Science and Engineering, Tianjin, China; ^2^Key Laboratory of Advanced Ceramics and Machining Technology (Ministry of Education), Tianjin University, Tianjin, China; ^3^Discipline of Chemistry, University of Newcastle, Callaghan, Newcastle, NSW, Australia; ^4^Key Laboratory of Advanced Energy Materials Chemistry (Ministry of Education), Nankai University, Tianjin, China; ^5^Research Institute of Tsinghua University in Shenzhen, Guangdong, China

**Keywords:** water splitting, cobalt sulfide, nanoparticle, graphene, HER/OER, composite

## Abstract

It is extremely meaningful to develop cheap, highly efficient, and stable bifunctional electrocatalysts for both hydrogen and oxygen evolution reactions (HER and OER) to promote large-scale application of water splitting technology. Herein, we reported the preparation of CoS_2_ nanoparticles supported on nitrogen-doped graphene (CoS_2_@N-GN) by one-step hydrothermal method and the enhanced electrochemical efficacy for catalyzing hydrogen and oxygen in water electrolysis. The CoS_2_@N-GN composites are composed of nitrogen-doped graphene and CoS_2_ nanocrystals with the average size of 73.5 nm. Benefitting from the improved electronic transfer and synergistic effect, the as-prepared CoS_2_@N-GN exhibits remarkable OER and HER performance in 1.0 M KOH, with overpotentials of 243 mV for OER and 204 mV for HER at 10 mA cm^−2^, and the corresponding Tafel slopes of 51.8 and 108 mV dec^−1^, respectively. Otherwise, the CoS_2_@N-GN hybrid also presents superior long-term catalytic durability. Moreover, an alkaline water splitting device assembled by CoS_2_@N-GN as both anode and cathode can achieve a low cell voltage of 1.53 V at 60 °C with a high faraday efficiency of 100% for overall water splitting. The tremendously enhanced electrochemical behaviors arise from favorable factors including small sized, homogenously dispersed novel CoS_2_ nanocrystals and coupling interaction with the underlying conductive nitrogen-doped graphene, which would provide insight into the rational design of transition metal chalcogenides for highly efficient and durable hydrogen and oxygen-involved electrocatalysis.

## Introduction

The large demand of clean and sustainable energy stimulated intensive research on the development of efficient and robust electrochemical energy conversion systems such as water splitting that can produce hydrogen and oxygen (Wang J. et al., 2016; Jia et al., 2017; Li H. et al., 2017; Zheng et al., 2017). It consists of two half reactions: the oxygen evolution reaction (OER) and hydrogen evolution reaction (HER) (Dong et al., 2015; Menezes et al., 2015; Sun et al., 2017; Han et al., 2018). However, slow reaction dynamics and high overpotential limit the wide application of water electrolyzer (Cheng et al., 2017a; Ma et al., 2017; Huang et al., 2018). High-performance electrocatalysts are urgently needed to reduce the overpotential and improve the energy efficiency (Fang et al., 2017; Yin et al., 2017; Shit et al., 2018). Currently, Pt-based materials and noble metal oxides (RuO_2_, IrO_2_) are the best catalysts for HER and OER (Han et al., 2014; Lee et al., 2015; Wang et al., 2015), respectively. However, high price and limited reserves restrict the wide application of these noble metal-based materials (Cheng et al., 2017b; Su et al., 2017). Moreover, these catalysts are still faced the problems of inferior long-term stability and unsatisfied bifunctional activity. Therefore, it is of great significance to design and develop high-abundant, high active and stable precious metal-free electrocatalysts (Forgie et al., 2010; Mccrory et al., 2015).

Recently, transition metal sulfides, especially cobalt sulfide, have attracted much research attention because of their earth-abundance, environmentally green, and significant chemical/electrochemical stability (Wang et al., 2009; Zheng et al., 2015; Han et al., 2017; Liu et al., 2017; Wu et al., 2017). However, the limited active sites and intrinsically low conductivity of traditional cobalt sulfide materials hinder their activity enhancement for catalyzing HER and OER (Liu et al., 2013). Therefore, the combination of metal sulfides and conductive materials is supposed to increase the electrical conductivity and meanwhile synergistically promoting the catalytic activity (Zhou et al., 2019). For example, Zn–Co-mixed sulfide nanostructure on carbon fiber paper has been synthesized for efficient rechargeable zinc–air batteries and water electrolysis (Wu et al., 2017); Co_9_S_8_ nanoparticles were anchored on nitrogen and sulfur dual-doped carbon nanosheets for bifunctional oxygen evolution and reduction reactions (Gulzar et al., 2017), CoS_x_/Ni_3_S_2_ heterostructure supported on nickel foam was developed as an efficient catalytic electrode for accelerating HER and OER (Wang et al., 2018). Previous investigations demonstrated that, among various cobalt sulfides (i.e., Co_3_S_4_, Co_9_S_8_, CoS_2_, etc.), the pyrite-type CoS_2_ exhibited the superior intrinsic performance owning to the abundant cobalt active sites and more proton-acceptor centers as a results of the unique crystal structure and S-rich nature (Kumar et al., 2018). Accordingly, it appears to be a smart strategy to design CoS_2_ nanostructure/conductive supported hybrid material to further optimize the HER and OER capability to replace the precious metal-based catalysts, thereby promoting the large-scale implementation of overall water splitting technologies (Wang J. et al., 2016; Xia et al., 2017).

In this work, we prepared the composite material of CoS_2_ uniformly supported on nitrogen-doped graphene (CoS_2_@N-GN) by one-step hydrothermal method and its application as bifunctional electrocatalytic catalyst for OER/HER and overall water-splitting in alkaline media. The average size of CoS_2_ is about 73.5 nm, homogeneously dispersed on the N-doped reduced graphene oxide nanosheet. Electrochemical tests reveal that the CoS_2_@N-GN hybrid exhibits superior catalytic performance than bare CoS_2_ and physically mixture sample (CoS_2_/N-GN), delivering low overpotentials of 243 and 204 mV at current density of 10 mA cm^−2^ for OER and HER, respectively. The *in-situ* grown composite electrode also presents remarkable long-term catalytic durability with negligible activity decay after 12 h period. Furthermore, the alkaline electrolyzer using CoS_2_@N-GN loaded on carbon fiber paper as both anode and cathode electrodes can achieve 10 mA cm^−2^ at a low cell voltage of 1.53 V at 60°C with a faradaic efficiency of 100% for overall water splitting. Our research will provide insights on the development of low cost and efficient hybrid electrocatalysts for next-generation energy conversion devices.

## Experimental section

### Regents and materials

Nitrogen doped graphene was purchased from Aladdin. Cobalt (II) acetate tetrahydrate (Co(AC)_2_·4H_2_O, 99.5%) and Potassium hydroxide (KOH, 99.99%) were purchased from Beijing Chemicals (Beijing, China). Carbon disulfide (CS_2_, 99%) and ethylenediamine (EN, 99%) were obtained from Tianjin Yuanli Chemical Co. Ltd. (Tianjin, China). The carbon paper (CP) was purchased from Phychemi Company Limited and used as the substrate of active substance. The deionized water (18.2 MΩ·cm^−1^) was obtained via Millipore, an ultrapure water system. High purity nitrogen (Air Product, purity 99.995%) gas was used to deaerate the 1 M KOH solution. All the reagents were of analytical grade and used as received without further purification.

### Materials synthesis

The CoS_2_@N-GN hybrid was synthesized by one step hydrothermal method. Typically, 30 mg of nitrogen doped graphene was put into a beaker with 100 mL and then 35 mL of deionized water was added followed by 30 min of ultrasonic agitation. Then, 0.4 mmol of Co(CH_3_COO)_2_·4H_2_O was added into the above mixed solution, followed by magnetic stirring under 800 rounds per minute (rpm) for 10 min. Then 0.16 mL of EN was added into the solution with magnetic stirring for 20 min. Afterwards, 0.16 mL of CS_2_ was dropped into the solution to form a uniform mixture after magnetic stirring for another 20 min. The above aqueous solution was then transferred into a 50 mL Teflon-lined stainless autoclave and then maintained at 200°C for 9 h. After cooling naturally to room temperature, the product was separated by centrifugal force, washed by distilled water for three times, and then lyophilized. The preparation of pure CoS_2_ was the same as the above route without adding 30 mg nitrogen doped graphene. The synthesis of physical mixture of CoS_2_ and N-doped graphene was achieved by mechanically mixed two components at mass ratio of 1:1.

### Materials characterization

The phase purity of the as-prepared samples was characterized by X-ray diffraction (XRD, Bruker/D8 Advanced with Cu Kα radiation) at a scanning rate of 2° min^−1^. The morphological micro-nanostructures were characterized by field-emission scanning electron microscopy (SEM, Hitachi S4800, 30 kV) and transmission electron microscopy (TEM, JEOL JEM-2100F, 200 kV) equipped with an energy-dispersive spectrometer (EDS). The Brunauer-Emmett-Teller (BET) specific surface area was determined by N_2_ adsorption/desorption isotherms at 77 K using the AutosorbiQ instrument (Quantachrome U.S.) with a 6 h outgas at 80°C. X-ray photoelectron spectroscopy (XPS) was conducted by using a Perkin Elmer PHI 1600 ECSA system.

### Electrocatalytic measurements

The electrocatalytic properties of the synthesized catalysts were tested on an IviumStat workstation using a three-electrode configuration. All the electrochemical data was performed in 1.0 M KOH electrolyte, which was saturated with high-purity N_2_ (Air Product, purity 99.995 %) for OER and HER for at least 30 min before each test and maintained under the corresponding atmosphere during the whole experiment. In addition, a saturated calomel electrode (SCE), and a platinum foil electrode were employed as reference electrode, and counter electrode. The linear sweeping voltammetry (LSV) of ORR and OER were scanning at a same scan rate of 5 mV s^−1^. The HER and OER potentials were corrected to the reversible hydrogen electrode (RHE) on the basis of following equation:

(1) $$\begin{array}{r} E\left(vs.\;RHE\right)=E\left(vs.\;SCE\right)+0.059\times pH+0.241V \end{array}$$

The electrochemical data of HER and OER was measured from −0.8 to −1.6 V vs. SCE, and 0.2 to 1.0 V vs. SCE, respectively. Electrochemical impedance spectra was carried out in a frequency range of from 100 to100 mHz at a potential of 0.6 V vs. RHE. The obtained HER and OER linear sweeping voltammetry (LSV) data was treated with iR-compensation according to the equation: E_c_ = E_m_ - I_m_ × R_s_, where E_c_, E_m_, I_m_, and R_s_ stand for compensated voltage, measured voltage, measured voltage and electrolyte resistance, respectively. The mass loading of synthesized electrocatalysts was about 1.5 mg cm^−2^. The RuO_2_ and Pt/C electrodes were also prepared for comparison with the same mass loading.

## Results and discussion

**Figure 1:**
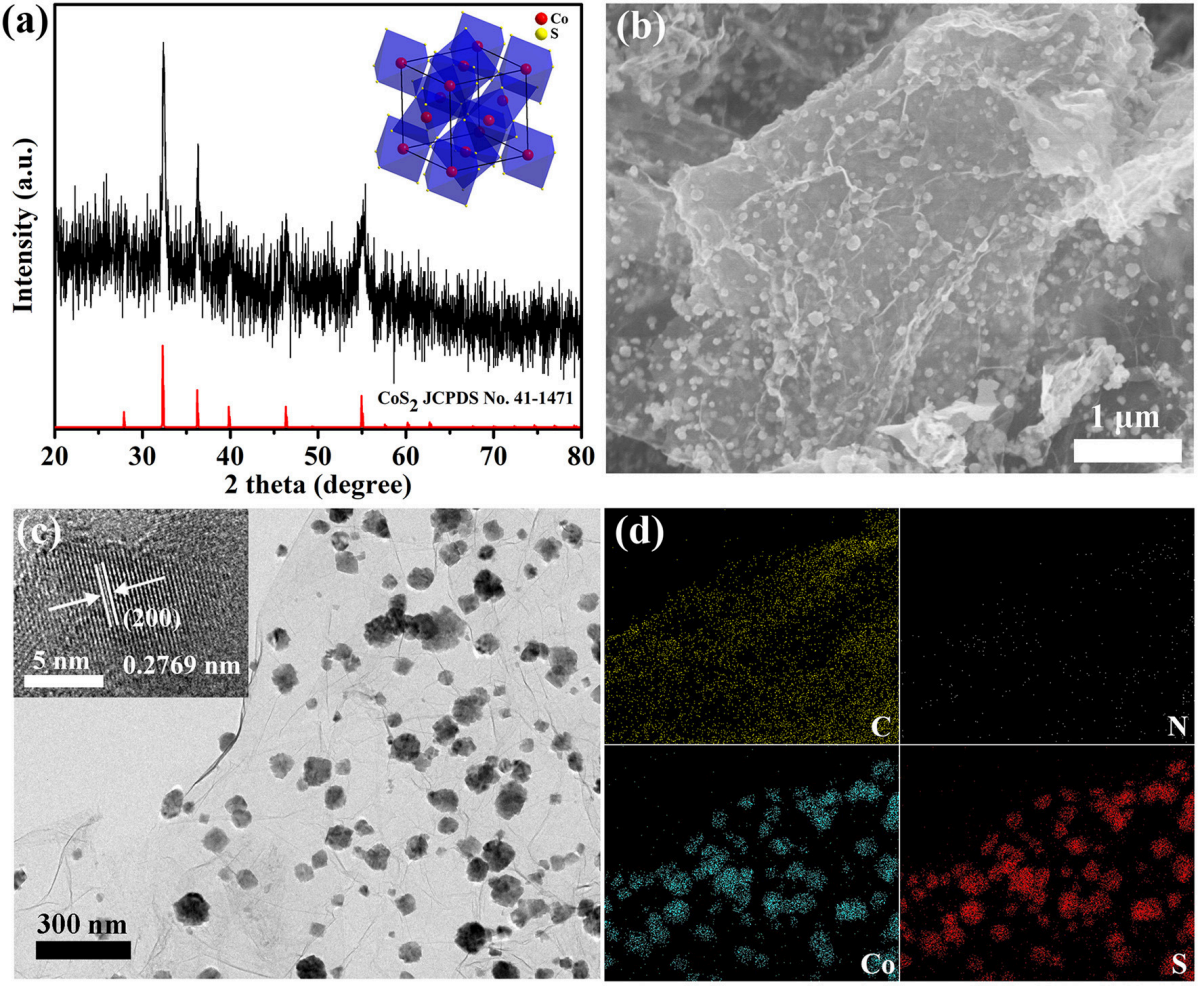
**(a)** XRD, **(b)** SEM, **(c)** TEM image, and **(d)** elemental mapping of CoS_2_@N-GN hybrid. Inset in **(a)** shows the crystal structure of pyrite-type CoS_2_. Inset in **(c)** shows the HRTEM image.

The synthetic procedure of the composite materials follows a facile, one-step, and hydrothermal method. As shown in Figure 1a, the power X-ray diffraction (XRD) pattern of CoS_2_@N-GN can be assigned to cubic CoS_2_ (JCPDS no. 41-1471), suggesting the successful formation of pyrite CoS_2_ phase in the hybrid. The morphological structures were characterized by scanning electron microscope (SEM) and transmission electron microscope (TEM) techniques. SEM image in Figure 1b revealed that CoS_2_ nanoparticles were uniformly anchored on nitrogen-doped graphene nanosheets. The average crystallize size is around 73.5 nm (Figure S1), which coincides with the Scherrer analysis based on the XRD pattern. The homogeneous distribution of CoS_2_ nanocrystals on graphene is further confirmed by the TEM imaging (Figure 1c). Moreover, in the high-resolution TEM (HRTEM) of inset in Figure 1c, an observed lattice spacing of 0.28 nm matches the (200) lattice plane of CoS_2_ (Ma et al., 2018), further proving the XRD analysis. Otherwise, the elemental mapping reveal the homogeneous dispersion of Co, S, C and N in the composite (Figure 1d), indicating the uniform distribution of CoS_2_ nanoparticles on the N-doping graphene substance. These observations collectively demonstrate the successful synthesis of CoS_2_@N-GN hybrid through stepwise controlled strategies. The Brunner–Emmet–Teller (BET) surface area of CoS_2_@N-GN hybrid was characterized to be 79.8 m^2^ g^−1^ (Figure S2). Thus, the CoS_2_@N-GN shows a significantly high BET area, which can provide more electroactive sites and facilitate electrolyte infiltration during the catalytic process (Li et al., 2018). The content of CoS_2_ in the hybrid is determined to be around 51 wt% (Figure S3) by thermal gravimetric analysis (TGA) (Liang et al., 2012).

**Figure 2:**
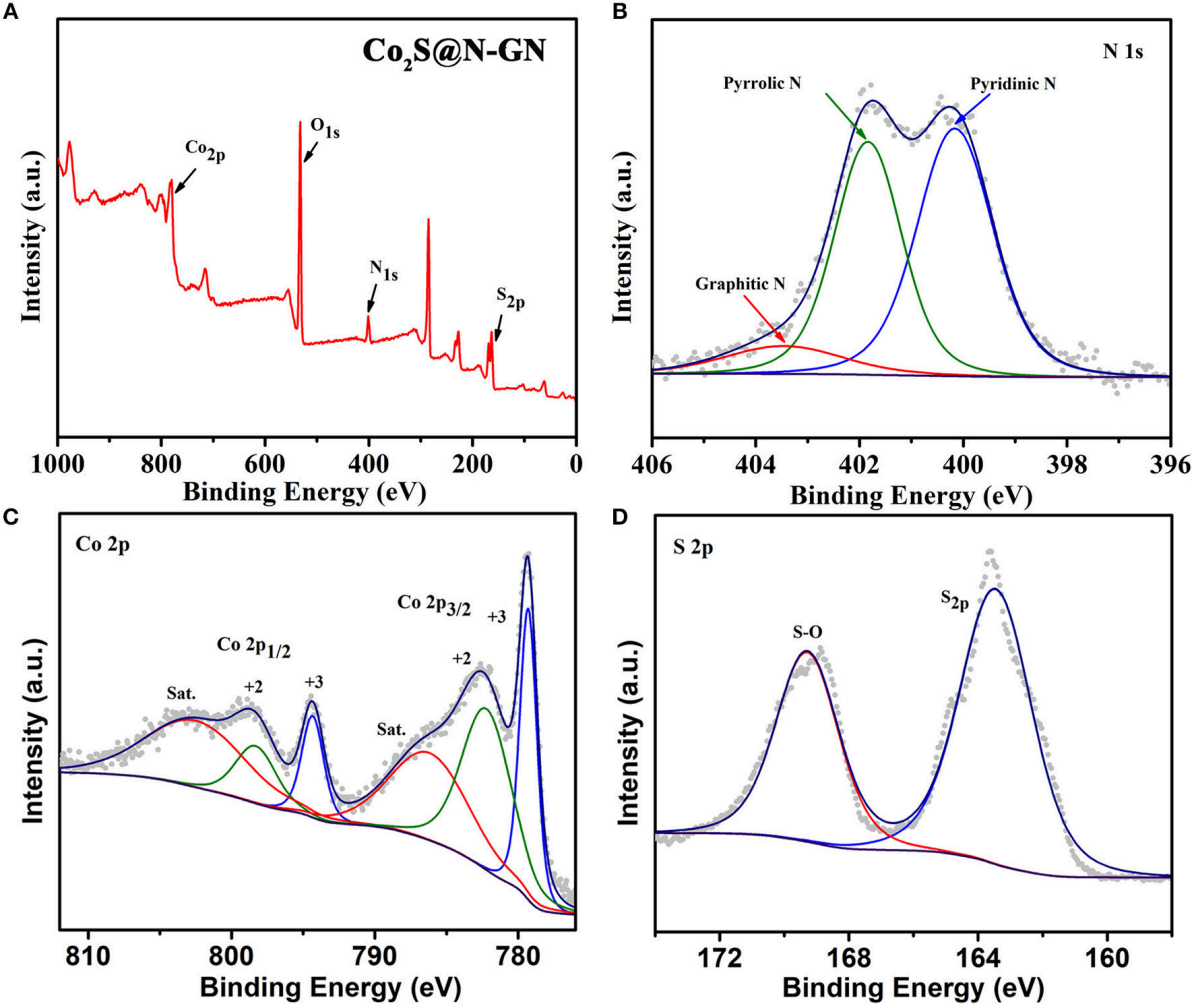
**(A)** XPS survey, **(B)** N 1s, **(c)** Co 2p, and **(D)** S 2p XPS spectra of the CoS_2_@N-GN hybrid.

The chemical valence states of N, Co, O and S in CoS_2_@N-GN were characterized by X-ray photoelectron spectroscopy (XPS, Figure 2). The XPS survey spectrum in Figure 2A shows the existence of C, N, Co, S, as well as O. The determined stoichiometric ratio is consistent with the result from energy-dispersive spectroscopy (Figure S4). The existence of O is due to exposure to air (Zhang et al., 2017). Additionally, the high-resolution N 1s spectrum (Figure 2B) can be deconvoluted into three sub-peaks located at 400.30 eV (pyridinic N), 402.20 eV (pyrrolic N), and 403.38 eV (graphitic N) (Fu et al., 2016; Li G. et al., 2016; Wang Z. et al., 2016). It is well known that doped N into carbon framework can potentially increase the active sites and thereby benefit the electrochemical catalytic activity enhancement (Ma et al., 2014; Li et al., 2018). The Co 2p spectra (Figure 2C) can be de-convoluted into six species, including pairs of fitting peaks for Co^2+^ and Co^3+^, and their shakeup satellites (Xiao et al., 2013; Ma et al., 2016; Zhu et al., 2016). The Co 2p_3/2_ peaks at 778.9 and 782.4 eV can be assigned to Co atoms in CoS_2_@N-GN and surface oxidized cobalt species coordinated with oxide or hydroxyl groups, respectively (Li C. et al., 2017). The main peak centered at 798.9 eV corresponds to spin–orbit characteristic peak of Co 2p_1/2_ in CoS_2_ compounds. For the high-resolution S 2p spectrum (Figure 2D), a weak doublet situated at 163.3 eV corresponds to S 2p. The peak located at 163.7 eV is assigned to the S 2p_1/2_ of S^2−^ ions that matched with metal ions (Du et al., 2015). The peak at 169.7 eV indicates the presence of a oxygen-sulfur (O-S) bond in the CoS_2_@N-GN compound, which may be ascribed to the surface oxidation, as confirmed by previous observations (Sivanantham et al., 2016).

**Figure 3:**
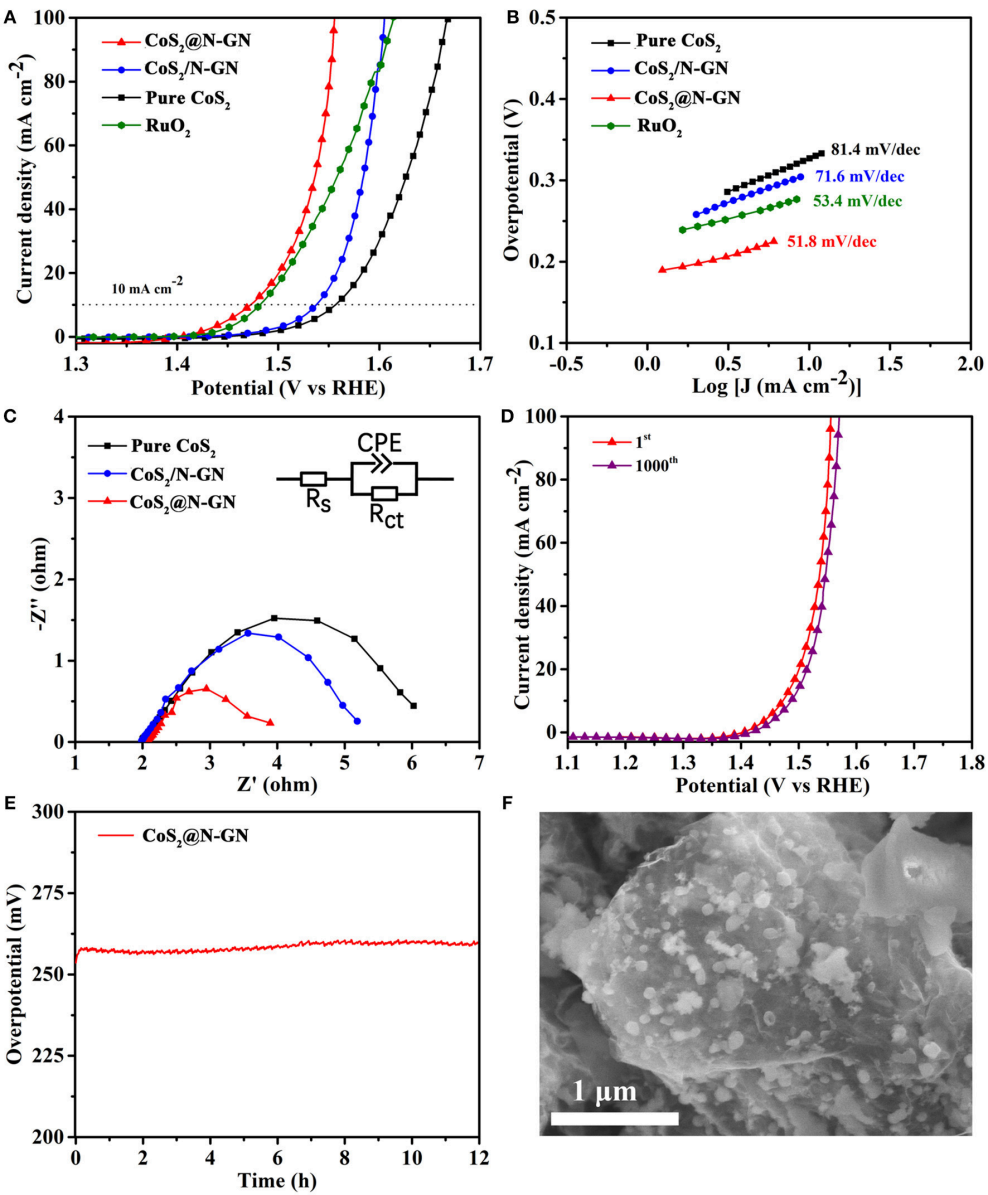
**(A)** OER LSVs of CoS_2_@N-GN, CoS_2_/N-GN, pure CoS_2_ in 1.0 M KOH at a scanning rate of 5 mV s^−1^. **(B)** Corresponding Tafel curves. **(C)** Nyquist plots of EIS at the potential of 1.56 V. **(D)** Polarization curves of CoS_2_@N-GN before and after 1000 cycles. **(E)** Chronopotentiometric response of CoS_2_@N-GN at a constant current density of 10 mA cm^−2^. **(F)** SEM image of the CoS_2_@N-GN catalyst after OER for 1000 cycles.

The electrocatalytic OER performance of synthesized CoS_2_@N-GN, together with CoS_2_/N-GN (the mechanical mixtures of pure CoS_2_ and nitrogen-doped graphene) and pure CoS_2_, was assessed by a three-electrode configuration in 1.0 M KOH. As shown in the polarization curves (Figure 3A and Table 1), CoS_2_@N-GN hybrid displays an overpotential of 204 mV to achieve an OER current density of 10 mA cm^−2^, which is much lower than those of CoS_2_/N-GN (278 mV) and pure CoS_2_ (297 mV), highlighting the superior OER activity of *in-situ* fabricated CoS_2_@N-GN. The fitted Tafel value of the CoS_2_@N-GN catalyst is 51.8 mV dec^−1^, which is lower than those of CoS_2_/N-GN (71.6 mV dec^−1^) and pure CoS_2_ (81.4 mV dec^−1^) (Figure 3B), indicating more favorable OER kinetics over the CoS_2_@N-GN surface. Otherwise, the corresponding charge transfer resistance (R_ct_) values of CoS_2_@N-GN, CoS_2_/N-GN and pure CoS_2_ are fitted to be 1.85, 3.2, 4.02 Ω, respectively (Figure 3C), suggesting that the efficient charge transfer contributes the superior activity of CoS_2_@N-GN electrode (Li P. et al., 2016). The presented OER performance parameters here are among the non-noble metal-based OER electrocatalysts reported in literatures (Table S1). In addition to good catalytic activity, long-term stability is also a critical factor in evaluating its practical performance. As we can see, the OER polarization curve of CoS_2_@N-GN after 1000 OER cycles at a scanning rate of 200 mV s^−1^ almost overlays the initial one (Figure 3D), evidencing that the activity is considerably maintained after the long-term continuous cycles. This is also corroborated by the chronopotentiometric response (Figure 3E), in which that the overpotential does not increase after 12 h period at an anodic current density of 10 mA cm^−2^. The SEM image of CoS_2_@N-GN further shows that the morphology is substantially unchanged after the 500 and 1000 OER cycles (Figure S5 and Figure 3f), signaling the remarkable structure durability of the composite catalyst. This may be mainly ascribed to the intrinsic crystal stability of CoS_2_ and firm attachment between the *in situ* grown sulfide nanostructures and the graphene support. The possible reaction mechanism of Co-based materials for catalyzing OER in alkaline media follows the sequence (Chen et al., 2015; Ma et al., 2018):

(2) $$\begin{array}{r} \text{Co}+2\text{O}{\text{H}}^{-}\to \text{Co}{\left(\text{OH}\right)}_{2}+2{\text{e}}^{-} \end{array}$$

(3) $$\begin{array}{r} 3\text{Co}{\left(\text{OH}\right)}_{2}+2\text{O}{\text{H}}^{-}\to \text{C}{\text{o}}_{3}{\text{O}}_{4}+4{\text{H}}_{2}\text{O} \end{array}$$

(4) $$\begin{array}{r} \text{C}{\text{o}}_{3}{\text{O}}_{4}+{\text{H}}_{2}\text{O}+\text{O}{\text{H}}^{-}\to 3\text{CoOOH}+{\text{e}}^{-} \end{array}$$

(5) $$\begin{array}{r} \text{CoOOH}+\text{O}{\text{H}}^{-}\to \text{Co}{\text{O}}_{2}+{\text{H}}_{2}\text{O}+{\text{e}}^{-} \end{array}$$

(6) $$\begin{array}{r} \text{Overall}:4\text{O}{\text{H}}^{-}\to 2{\text{H}}_{2}\text{O}+{\text{O}}_{2}+4{\text{e}}^{-} \end{array}$$

As shown in Figure S6a, after 1000 OER cycles, it can be clearly seen that the proportion of Co^3+^ is significantly increased. Meanwhile, the proportion of S-O is also significantly increased (Figure S6b), suggesting the considerable oxidation, consistent with previous observations (Han et al., 2018; Ma et al., 2018).

**Table 1:** Summary of the electrochemical activities of CoS_2_@N-GN, CoS_2_/N-GN and pure CoS_2_ electrodes.

**Catalysts**	**Reaction**	**tafel slope (mA dec^−1^)**	**Overpotential @10 mA cm^−2^ (mV)**	**R_ct_ (Ω)**	**C_dl_ (mF cm^−2^)**
CoS_2_@N-GN	OER	51.8	243	1.85	76.7
	HER	108.5	204	
CoS_2_/N-GN	OER	71.6	307	3.2	20.1
	HER	139.1	278	
pure CoS_2_	OER	81.4	327	4.02	13.6
	HER	144.7	297	

**Figure 4:**
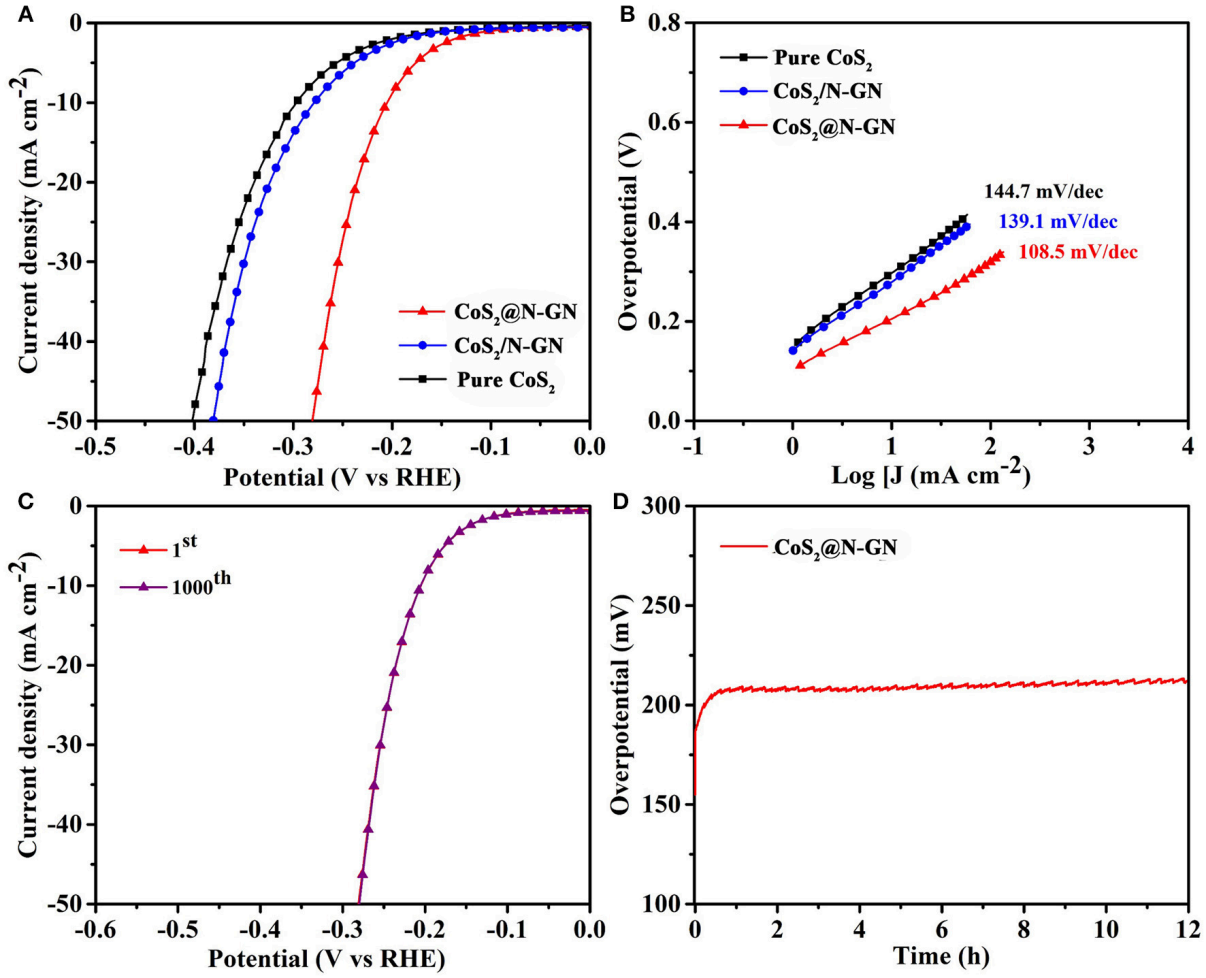
**(A)** HER LSV curves of CoS_2_@N-GN, CoS_2_/N-GN, pure CoS_2_ in 1.0 M KOH at a scanning rate of 5 mV s^−1^. **(B)** Corresponding Tafel slopes. **(C)** HER polarization curves of CoS_2_@N-GN before and after 1,000 cycles. **(D)** Chronopotentiometric response of CoS_2_@N-GN at a constant current density of 10 mA cm^−2^.

In the context of developing bifunctional catalysts for overall water oxidation, the HER performance of synthesized samples was tested in the same electrolyte. All the linear sweeping voltammetry (LSVs) measurements were collected at a scan rate of 5 mV s^−1^ while the electrolyte is saturated with N_2_ during the experiment. From the HER LSVs in Figure 4A, it is clear that the pure CoS_2_ shows weak electrocatalytic HER activity. The *in-situ* fabricated CoS_2_@N-GN achieves a current density of 10 mA cm^−2^ at an overpotential of 204 mV, which is much lower than that of physical mixture of CoS_2_/N-GN (278 mV) and pure CoS_2_ (297 mV).

The corresponding Tafel slopes of N-GP@CoS_2_, N-GP/CoS_2_, and pure CoS_2_ are 108.5, 139.1, 144.7 mV dec^−1^, respectively, which is comparable to those of transition metal sulfide catalysts reported recently (Figure 4B) (Liang et al., 2016; Sivanantham et al., 2016). In particular, N-GP@CoS_2_ shows the lowest Tafel slope of 108.5 mV dec^−1^ among three synthesized catalysts. The disparity in Tafel slopes further corroborates the advantage of the synergistic effect between N-GN and CoS_2_. The double-layer capacitance (C_dl_) values are determined to be 76.7, 20.1 and 13.6 mF cm^−2^ for CoS_2_@N-GN, CoS_2_/N-GN, and pure CoS_2_ (Figure S7), demonstrated that the CoS_2_@N-GN could provide larger electrochemical active area for electrolyte soakage and water adsorption, which is another favorable factor in enhancing the electrocatalytic capability. The HER typically occurs through two reactions in alkaline solution (Li H. et al., 2017; Ma et al., 2018) Volmer reaction (H_2_O + e^−^ + M → M-H${}_{\text{ad}}^{*}$ + OH^−^, where H${}_{\text{ad}}^{*}$ is a reactive intermediate and M is the catalytically active site) and Heyrovsky reaction (H_2_O + M-H${}_{\text{ad}}^{*}$ + e^−^ → M + H_2_ + OH^−^). The key HER performance parameters presented here are among the best non-noble metal HER electrocatalysts reported in literatures (Table S2). Moreover, after 1000 CV cycles, the polarization curve of CoS_2_@N-GN is remarkably maintained in comparison with the initial state (Figure 4C). The morphology of CoS_2_@N-GN electrode was still essentially preserved (Figure S8), further confirming the structural durability, which is mainly due to the intrinsic stability of CoS_2_ and the strong chemical interaction between two components. In a continuous polarization period of 12 h (Figure 4D), the HER current retention of CoS_2_@N-GN again confirms its remarkable long-term stability for catalyzing the HER in alkaline media.

**Figure 5:**
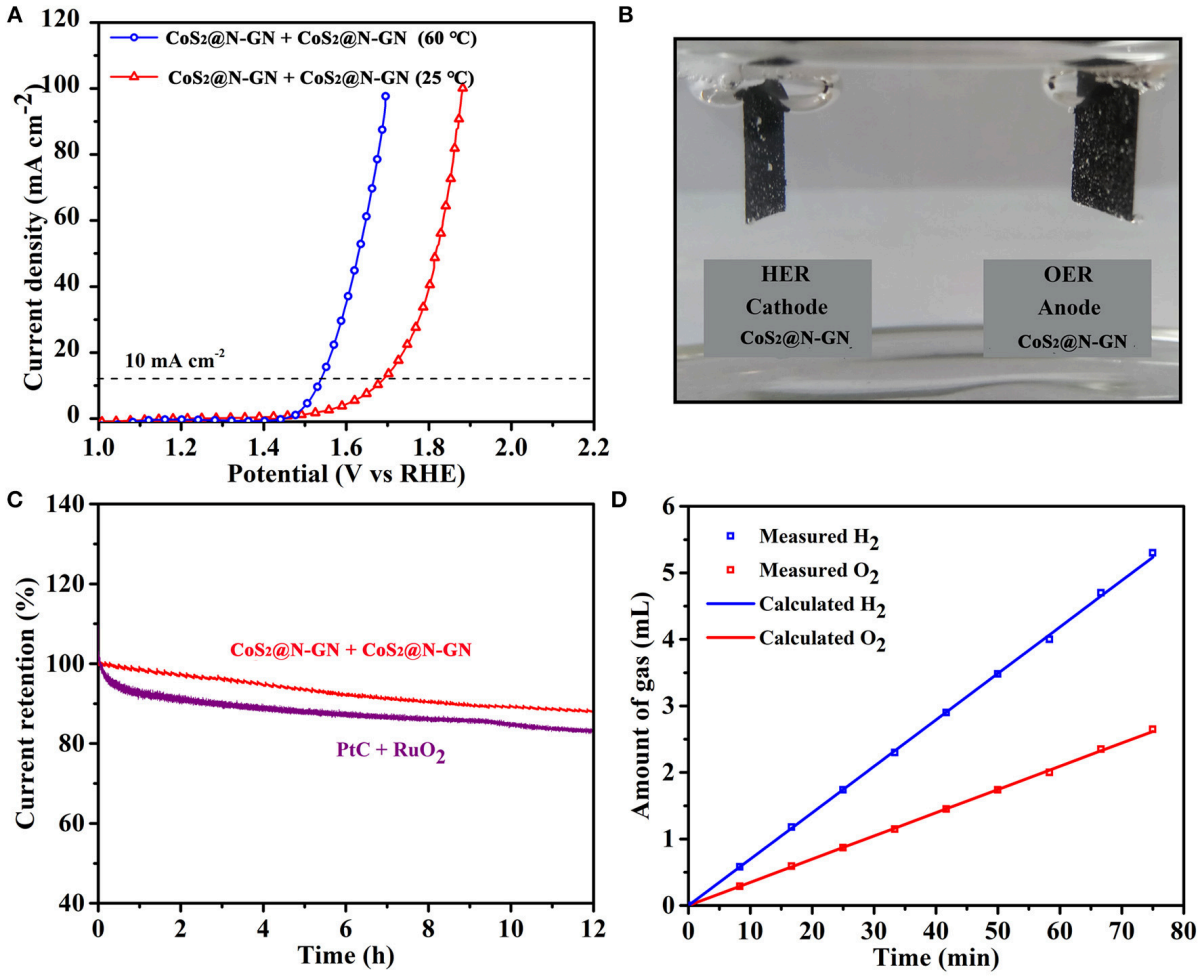
**(A)** Overall water splitting performance of N-GP@CoS_2_ at different temperatures. **(B)** The photograph of gas evolution at 10 mA cm^−2^. **(C)** Chronoamperometric response of two overall water splitting systems at 1.9 V. **(D)** The volumes of H_2_ and O_2_ experimentally measured and theoretically calculated vs. time at 10 mA cm^−2^.

As shown in Figure 5A, to further investigate its practical application for overall water-splitting, CoS_2_@N-GN electrode was applied as both anode and cathode to assemble an electrochemical device, which presents low cell voltages of 1.70 V at 25°C and 1.53 V at 60°C to achieve 10 mA cm^−2^ in 1 M KOH. The generated oxygen and hydrogen bubbles on both anode and cathode surface can be obviously seen at 10 mA cm^−2^ (Figure 5B). In addition, the stability of the electrolyzer device was tested with sustained polarization for 12 h at 1.90 V. The activity degradation of CoS_2_@N-GN (8.1%) is even lower than that of the combined catalyst of precious Pt/C + RuO_2_ (17.6%, Figure 5C), further demonstrating the considerable durability of the CoS_2_@N-GN electrode in a practical water splitting device. As displayed in Figure 5D, the amounts of collected H_2_ and O_2_ match well with the calculated values at a constant current density of 10 mA cm^−2^, which shows advanced concept of efficient energy conversion from electric energy to chemical fuel gas. The relationship between time and gas quantity is calculated according to the following formula:

$$\begin{array}{l} \frac{I\;*\text{ t}}{4\;*\;e}=\frac{V\;*\;N_{A}}{22.4} \end{array}$$

*I, t, e, V and N*_*A*_ are current (mA), time (s), 1.6 × 10^−19^ C, volume (L) and avogadro constant (6.02 × 10^23^).

## Conclusion

In summary, a novel CoS_2_@N-GN hybrid was successfully prepared through a facile and one-step hydrothermal strategy. Compared with CoS_2_/N-GN and pure CoS_2_ catalyst, CoS_2_@N-GN hybrid exhibits remarkable overall electrocatalytic activity toward OER and HER in alkaline electrolyte as well as the enhanced long-term stability. Moreover, the CoS_2_@N-GN enables the assembled water splitting device with low cell voltage, high efficiency and prolonged operational life. The remarkable electrochemical properties are attributed to the high intrinsic activity of CoS_2_, efficient electron transfer provided by N-doped graphene and the synergetic coupling interaction between two components. This work establishes the low cost and earth-abundant metal sulfide-based composite catalyst as a promising and high performance functional electrode for promoting large-scale water electrolyzer technologies.

## Author contributions

WZ conducted the experiments and helped write the manuscript. XM helped with operating the experiments and data analysis. CZ and YD interpreted the results. XH, TM, and WH supervised the research. All authors read and approved the final manuscript.

### Conflict of interest statement

The authors declare that the research was conducted in the absence of any commercial or financial relationships that could be construed as a potential conflict of interest.

## References

[B1] Chen P. Z. Xu K. Fang Z. W. Tong Y. Wu J. C. Lu X. L.. (2015). Metallic Co_4_N porous nanowire arrays activated by surface oxidation as electrocatalysts for the oxygen evolution reaction. Angew. Chem. Int. Ed. 54,14710–14714. 10.1002/anie.201506480 26437900

[B2] Cheng H. Li M. L. Su C. Y. Li N. Liu Z. Q. (2017a). Cu-Co bimetallic oxide quantum dot decorated nitrogen-doped carbon nanotubes: a high-efficiency bifunctional oxygen electrode for zn-air batteries. Adv. Funct. Mater. 27:1701833 10.1002/adfm.201701833

[B3] Cheng H. Su C.-Y. Tan Z.-Y. Tai S.-Z. Liu Z.-Q. (2017b). Interacting ZnCo_2_O_4_ and Au nanodots on carbon nanotubes as highly efficient water oxidation electrocatalyst. J. Power Sources 357, 1–10. 10.1016/j.jpowsour.2017.04.091

[B4] Dong U. L. Park M. G. Park H. W. Min H. S. Ismayilov V. Ahmed R. (2015). Highly active Co-doped LaMnO_3_ perovskite oxide and N-doped carbon nanotube hybrid bi-functional catalyst for rechargeable zinc–air batteries. Electrochem. Commun. 60, 38–41. 10.1016/j.elecom.2015.08.001

[B5] Du Y. Zhu X. Zhou X. Hu L. Dai Z. Bao J. (2015). Co_3_S_4_ porous nanosheets embedded in graphene sheets as high-performance anode materials for lithium and sodium storage. J. Mater. Chem. A 3, 6787–6791. 10.1039/c5ta00621j

[B6] Fang L. Li W. Guan Y. Feng Y. Zhang H. Wang S. (2017). Tuning Unique Peapod Like Co(S_x_Se_1−−x_)_2_ nanoparticles for efficient overall water splitting. Adv. Funct. Mater. 27:1701008 10.1002/adfm.201701008

[B7] Forgie R. Bugosh G. Neyerlin K. C. Liu Z. Strasser P. (2010). Bimetallic Ru electrocatalysts for the OER and electrolytic water splitting in acidic media. Electrochem. Solid ST 13, 36–39. 10.1149/1.3290735

[B8] Fu J. Hassan F. M. Li J. Lee D. U. Ghannoum A. R. Lui G. (2016). Flexible rechargeable zinc-air batteries through morphological emulation of human hair array. Adv. Mater. 28, 6421–6428. 10.1002/adma.201600762 27197721

[B9] Gulzar A. Xu J. Yang P. He F. Xu L. (2017). Upconversion processes: versatile biological applications and biosafety. Nanoscale 9, 12248–12282. 10.1039/c7nr01836c 28829477

[B10] Han X. Cheng F. Zhang T. Yang J. Hu Y. Chen J. (2014). Hydrogenated uniform Pt Clusters supported on porous CaMnO_3_ as a bifunctional electrocatalyst for enhanced oxygen reduction and evolution. Adv. Mater. 26, 2047–2051. 10.1002/adma.201304867 24818256

[B11] Han X. Wu X. Deng Y. Liu J. Lu J. Zhong C. (2018). Ultrafine Pt nanoparticle-decorated pyrite-Type CoS_2_ nanosheet arrays coated on carbon cloth as a bifunctional electrode for overall water splitting. Adv. Energy Mater. 8:1800935 10.1002/aenm.201800935

[B12] Han X. Wu X. Zhong C. Deng Y. Zhao N. Hu W. (2017). NiCo_2_S_4_ nanocrystals anchored on nitrogen-doped carbon nanotubes as a highly efficient bifunctional electrocatalyst for rechargeable zinc-air batteries. Nano Energy 31, 541–550. 10.1016/j.nanoen.2016.12.008

[B13] Huang C. Ouyang T. Zou Y. Li N. Liu Z.-Q. (2018). Ultrathin NiCo_2_P_x_ nanosheets strongly coupled with CNTs as efficient and robust electrocatalysts for overall water splitting. J. Mater. Chem. A. 6, 7420–7427. 10.1039/C7TA11364A

[B14] Jia Y. Zhang L. Gao G. Chen H. Wang B. Zhou J.. (2017). A Heterostructure coupling of exfoliated Ni–Fe hydroxide nanosheet and defective graphene as a bifunctional electrocatalyst for overall water splitting. Adv. Mater. 29:1700017. 10.1002/adma.201700017 28256771

[B15] Kumar N. Su W. Veselý M. Weckhuysen B. M. Pollard A. J. Wain A. J. (2018). Nanoscale chemical imaging of solid-liquid interfaces using tip-enhanced Raman spectroscopy. Nanoscale 10, 1815–1824. 10.1039/c7nr08257f 29308817

[B16] Lee Y. Jin S. May K. J. Perry E. E. Yang S. H. (2015). Synthesis and activities of rutile IrO_2_ and RuO_2_ nanoparticles for oxygen evolution in acid and alkaline solutions. J. Phys. Chem. Lett. 3, 399–404. 10.1021/jz2016507 26285858

[B17] Li C. Du Y. Wang D. Yin S. Tu W. Chen Z. (2017). Unique P-Co-N surface bonding states constructed on g-C_3_N_4_ nanosheets for drastically enhanced photocatalytic activity of H_2_ evolution. Adv. Funct. Mater. 27:1604328 10.1002/adfm.201604328

[B18] Li G. Wang X. Fu J. Li J. Park M. G. Zhang Y.. (2016). Pomegranate-inspired design of highly active and durable bifunctional electrocatalysts for rechargeable metal-air batteries. Angew. Chem. Int. Ed. 55, 4977–4982. 10.1002/anie.201600750 26970076

[B19] Li H. Wen P. Li Q. Dun C. Xing J. Lu C. (2017). Earth-abundant iron diboride (FeB_2_) nanoparticles as highly active bifunctional electrocatalysts for overall water splitting. Adv. Energy Mater. 7:1700513 10.1002/aenm.201700513

[B20] Li P. Yang Z. Shen J. Nie H. Cai Q. Li L.. (2016). Subnanometer molybdenum sulfide on carbon nanotubes as a highly active and stable electrocatalyst for hydrogen evolution reaction. ACS Appl. Mater. Interfaces 8, 3543–3550. 10.1021/acsami.5b08816 26765150

[B21] Li Y. Zhong C. Liu J. Zeng X. Qu S. Han X. (2018). Atomically thin mesoporous Co_3_O_4_ layers strongly coupled with N-rGO nanosheets as high-performance bifunctional catalysts for 1D knittable zinc-air batteries. Adv. Mater. 30:1703657 10.1002/adma.201703657 29210114

[B22] Liang Y. Liu Q. Luo Y. Sun X. He Y. Asiri A. M. (2016). Zn_0.76_Co_0.24_S/CoS_2_ nanowires array for efficient electrochemical splitting of water. Electrochim. Acta 190, 360–364. 10.1016/j.electacta.2015.12.153

[B23] Liang Y. Wang H. Zhou J. Li Y. Wang J. Regier T.. (2012). Covalent hybrid of spinel manganese-cobalt oxide and graphene as advanced oxygen reduction electrocatalysts. J. Am. Chem. Soc. 134, 3517–3523. 10.1021/ja210924t 22280461

[B24] Liu J. Wang J. Zhang B. Ruan Y. Lv L. Ji X.. (2017). Hierarchical NiCo2S4@NiFe LDH Heterostructures Supported on Nickel Foam for Enhanced Overall-Water-Splitting Activity. ACS Appl. Mater. Interfaces 9, 15364–15372. 10.1021/acsami.7b00019 28332812

[B25] Liu Q. Jin J. Zhang J. (2013). NiCo_2_S_4_@graphene as a bifunctional electrocatalyst for oxygen reduction and evolution reactions. ACS Appl. Mater. Interfaces 5, 5002–5008. 10.1021/am4007897 23662625

[B26] Ma F. X. Yu L. Xu C. Y. Lou X. W. (2016). Self-supported formation of hierarchical NiCo_2_O_4_ tetragonal microtubes with enhanced electrochemical properties. Energy Environ. Sci. 9, 862–866. 10.1039/c5ee03772g

[B27] Ma T. Y. Dai S. Jaroniec M. Qiao S. Z. (2014). Graphitic carbon nitride nanosheet–carbon nanotube three-dimensional porous composites as high-performance oxygen evolution electrocatalysts. Angew. Chem. Int. Ed. 53, 7281–7285. 10.1002/anie.201403946 24888731

[B28] Ma X. Wang J. Liu D. Kong R. M. Hao S. Du G. (2017). Hydrazine-assisted electrolytic hydrogen production: CoS_2_ nanoarray as a superior bifunctional electrocatalyst. New J. Chem. 41, 4754–4757. 10.1039/C7NJ00326A

[B29] Ma X. Zhang W. Deng Y. Zhong C. Hu W. Han X. (2018). Phase and composition controlled synthesis of cobalt sulfide hollow nanospheres for electrocatalytic water splitting. Nanoscale 10, 4816–4824. 10.1039/c7nr09424h 29473086

[B30] Mccrory C. C. L. Jung S. Ferrer I. M. Chatman S. M. Peters J. C. Jaramillo T. F. (2015). Benchmarking hydrogen evolving reaction and oxygen evolving reaction electrocatalysts for solar water splitting devices. J. Am. Chem. Soc. 137, 4347–4357. 10.1021/ja510442p 25668483

[B31] Menezes P. W. Indra A. Littlewood P. Schwarze M. Göbel C. Schomäcker R.. (2015). Nanostructured manganese oxides as highly active water oxidation catalysts: a boost from manganese precursor chemistry. Chemsuschem 7, 2202–2211. 10.1002/cssc.201402169 25044528

[B32] Shit S. Chhetri S. Jang W. Murmu N. C. Koo H. Samanta P.. (2018). Cobalt sulfide/nickel sulfide heterostructure directly grown on nickel foam: an efficient and durable electrocatalyst for overall water splitting application. ACS Appl. Mater. Interfaces 10, 27712–27722. 10.1021/acsami.8b04223 30044090

[B33] Sivanantham A. Ganesan P. Shanmugam S. (2016). Hierarchical NiCo_2_S_4_ nanowire arrays supported on ni foam: an efficient and durable bifunctional electrocatalyst for oxygen and hydrogen evolution reactions. Adv. Funct. Mater. 26, 4661–4672. 10.1002/adfm.201600566

[B34] Su C. Y. Cheng H. Li W. Liu Z. Q. Li N. Hou Z. (2017). Atomic modulation of FeCo–nitrogen–carbon bifunctional oxygen electrodes for rechargeable and flexible all soli-state zinc-air battery. Adv. Energy Mater. 7:1602420 10.1002/aenm.201602420

[B35] Sun Y. Hang L. Shen Q. Zhang T. Li H. Zhang X.. (2017). Mo doped Ni_2_P nanowire arrays: an efficient electrocatalyst for the hydrogen evolution reaction with enhanced activity at all pH values. Nanoscale 9, 16674–16679. 10.1039/c7nr03515b 28820219

[B36] Wang H. Lee H. W. Deng Y. Lu Z. Hsu P. C. Liu Y.. (2015). Bifunctional non-noble metal oxide nanoparticle electrocatalysts through lithium-induced conversion for overall water splitting. Nat. Commun. 6, 7261–7269. 10.1038/ncomms8261 26099250 PMC4557299

[B37] Wang J. Zhong H. Wang Z. Meng F. Zhang X. (2016). Integrated three-dimensional carbon paper/carbon tubes/cobalt-sulfide sheets as an efficient electrode for overall water splitting. ACS Nano 10, 2342–2348. 10.1021/acsnano.5b07126 26783885

[B38] Wang K. Liu X. K. Chen X. H. Yu D. G. Yang Y. Y. Liu P. (2018). Electrospun hydrophilic janus nanocomposites for the rapid onset of therapeutic action of helicid. ACS Appl. Mater. Interfaces 10, 2859–2867. 10.1021/acsami.7b17663 29272099

[B39] Wang X. Z. Liu S. S. Sun Y. Wu J. Y. Zhou Y. L. Zhang J. H. (2009). Beta-cypermethrin impairs reproductive function in male mice by inducing oxidative stress. Theriogenology 72, 599–611. 10.1016/j.theriogenology.2009.04.016 19500828

[B40] Wang Z. Li B. Ge X. Goh F. W. T. Zhang X. Du G.. (2016). Co@Co_3_O_4_@PPD Core@bishell nanoparticle-based composite as an efficient electrocatalyst for oxygen reduction reaction. Small 12, 2580–2587. 10.1002/smll.201503694 27031907

[B41] Wu X. Han X. Ma X. Zhang W. Deng Y. Zhong C.. (2017). Morphology-controllable synthesis of Zn-Co-mixed sulfide nanostructures on carbon fiber paper toward efficient rechargeable Zinc-air batteries and water electrolysis. ACS Appl. Mater. Interfaces 9, 12574–12583. 10.1021/acsami.6b16602 28319373

[B42] Xia C. Liang H. Zhu J. Schwingenschlögl U. Alshareef H. N. (2017). Active edge sites engineering in nickel cobalt selenide solid solutions for highly efficient hydrogen evolution. Adv. Energy Mater. 7:1602089 10.1002/aenm.201602089

[B43] Xiao J. Kuang Q. Yang S. Xiao F. Wang S. Guo L. (2013). Surface structure dependent electrocatalytic activity of Co_3_O_4_anchored on graphene sheets toward oxygen reduction reaction. Sci. Rep. 3, 2300–2308. 10.1038/srep02300 23892418 PMC3725507

[B44] Yin J. Li Y. Lv F. Lu M. Sun K. Wang W.. (2017). Oxygen vacancies dominated NiS_2_/CoS_2_ interface porous nanowires for portable Zn–air batteries driven water splitting devices. Adv. Mater. 29:1704681. 10.1002/adma.201704681 29239518

[B45] Zhang G. Wang P. Lu W. T. Wang C. Y. Li Y. K. Ding C.. (2017). Co nanoparticles/Co, N, S tri-doped graphene templated from in-situ-formed Co, S Co-doped g-C_3_N_4_ as an active bifunctional electrocatalyst for overall water splitting. ACS Appl. Mater. Interfaces 9, 28566–28576. 10.1021/acsami.7b08138 28796474

[B46] Zheng G. Peng Z. Jia D. Al-Enizi A. M. Elzatahry A. A. (2015). From water oxidation to reduction: Homologous Ni-Co based nanowires as complementary water splitting electrocatalysts. Adv. Energy Mater. 5:1402031 10.1002/aenm.201402031

[B47] Zheng M. Ding Y. Yu L. Du X. Zhao Y. (2017). *In Situ* grown pristine cobalt sulfide as bifunctional photocatalyst for hydrogen and oxygen evolution. Adv. Funct. Mater. 27:1605846 10.1002/adfm.201605846

[B48] Zhou S. Zhou Q.-X. Su H. Wang Y. Dong Z. Dai X.. (2019). Hybrid of Fe_3_C@N, S co-doped carbon nanotubes coated porous carbon derived from metal organic frameworks as an efficient catalyst towards oxygen reduction. J. Colloid Inter. Sci. 533, 311–318. 10.1016/j.jcis.2018.06.091 30170281

[B49] Zhu Y. P. Ma T. Y. Jaroniec M. Qiao S. Z. (2016). Self-templating synthesis of hollow Co_3_O_4_ microtube arrays for highly efficient water electrolysis. Angew. Chem. Int. Ed. 56, 1324–1328. 10.1002/anie.201610413 27900829

