# Supplementary material for: Pyrite-Type CoS2 Nanoparticles Supported on Nitrogen-Doped Graphene for Enhanced Water Splitting
Source: Front Chem. 2018 Nov 21;6:569. doi: 10.3389/fchem.2018.00569 (PMC6258795; doi:10.3389/fchem.2018.00569)
Supplement: Supplementary file 1 [file Table_1.DOCX]

**Supporting Information**

Pyrite-Type CoS_2_ Nanoparticles Supported on Nitrogen-Doped Graphene for Enhanced Water Splitting

Wei Zhang,^1^ XiaoyaMa,^1^ Cheng Zhong,^1,2^ Tianyi Ma,^*,3^ Yida Deng^1,2^ Wenbin Hu^1,2^ and Xiaopeng Han,^*,1,2,4^

^1^Tianjin Key Laboratory of Composite and Functional Materials, School of Materials Science and Engineering, ^2^Key Laboratory of Advanced Ceramics and Machining Technology (Ministry of Education), Tianjin University, Tianjin 300072, China; ^3^Discipline of Chemistry, University of Newcastle, Callaghan, Newcastle, NSW 2308, Australia; ^4^Key Laboratory of Advanced Energy Materials Chemistry (Ministry of Education), Nankai University, Tianjin 300071, China.

**
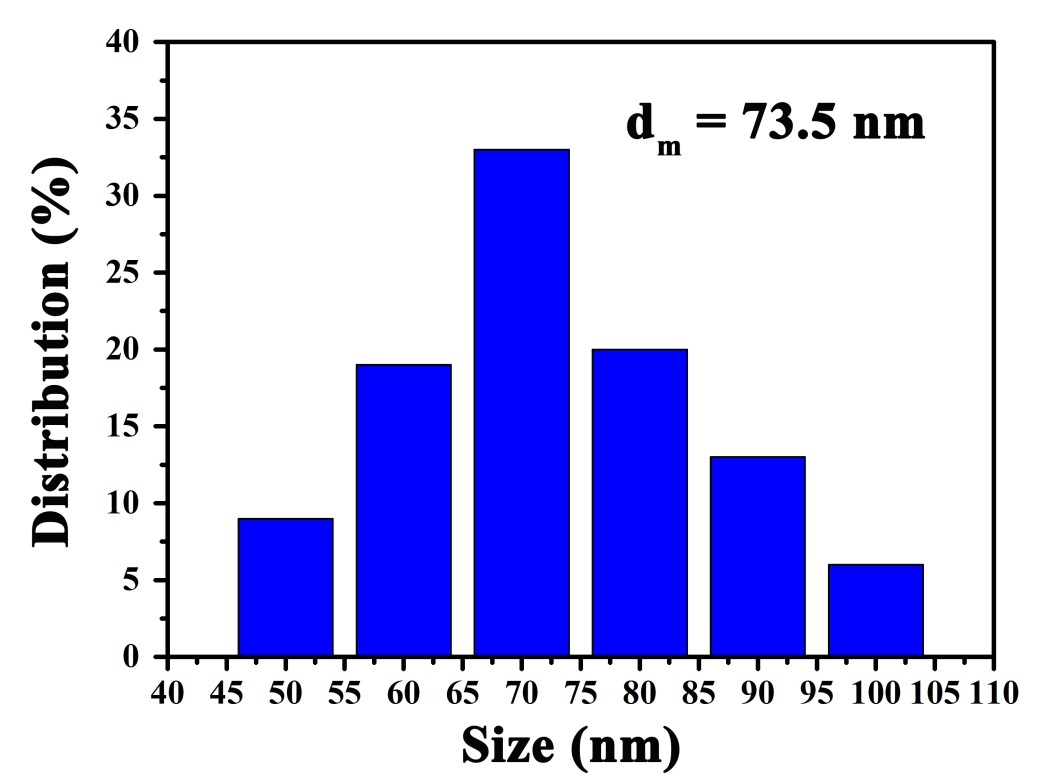
**

**Figure S1.** The particle size distribution of CoS_2_ in CoS_2_@N-GN hybrid.

**
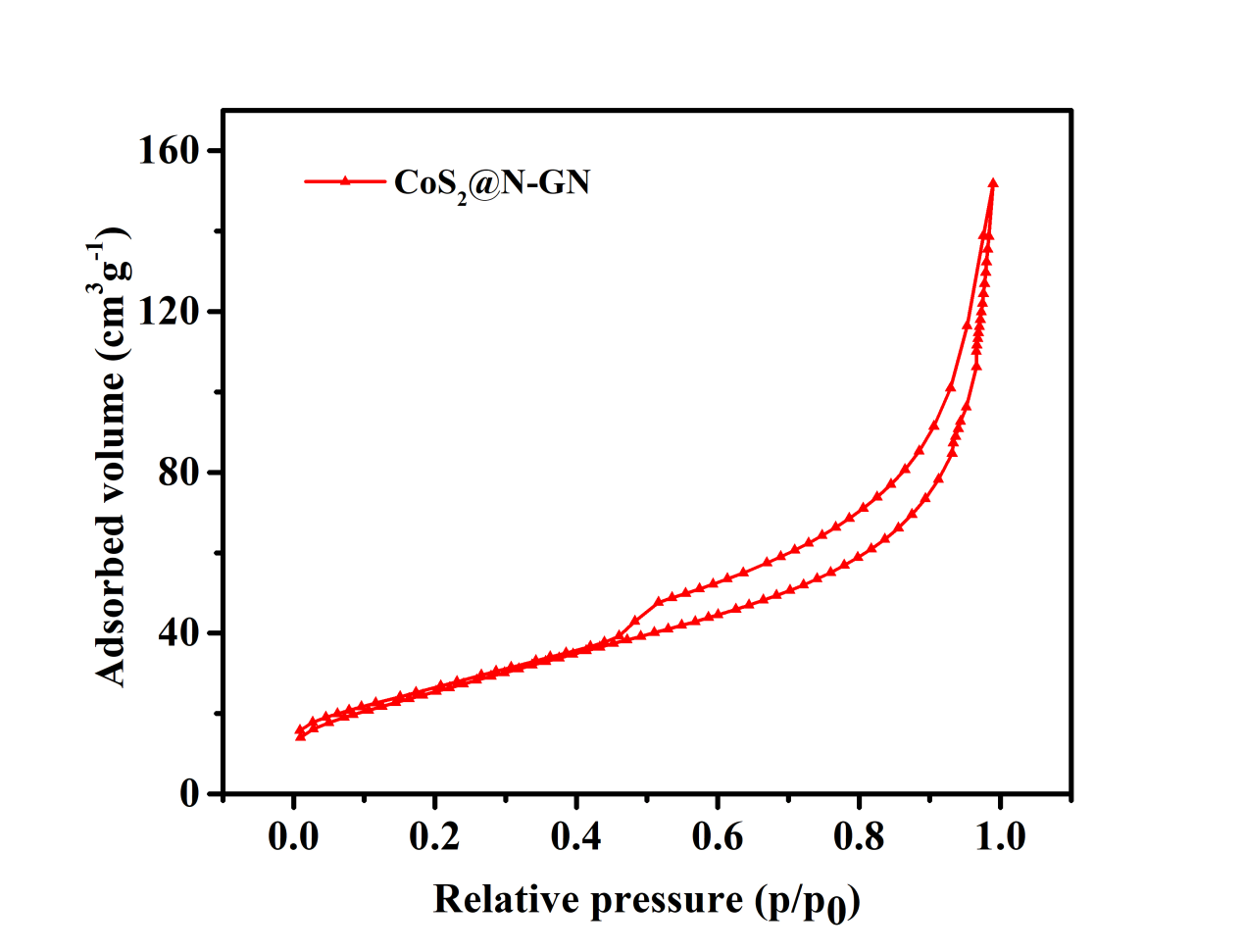
**

**Figure S2.** BET curve of CoS_2_@N-GN hybrid.


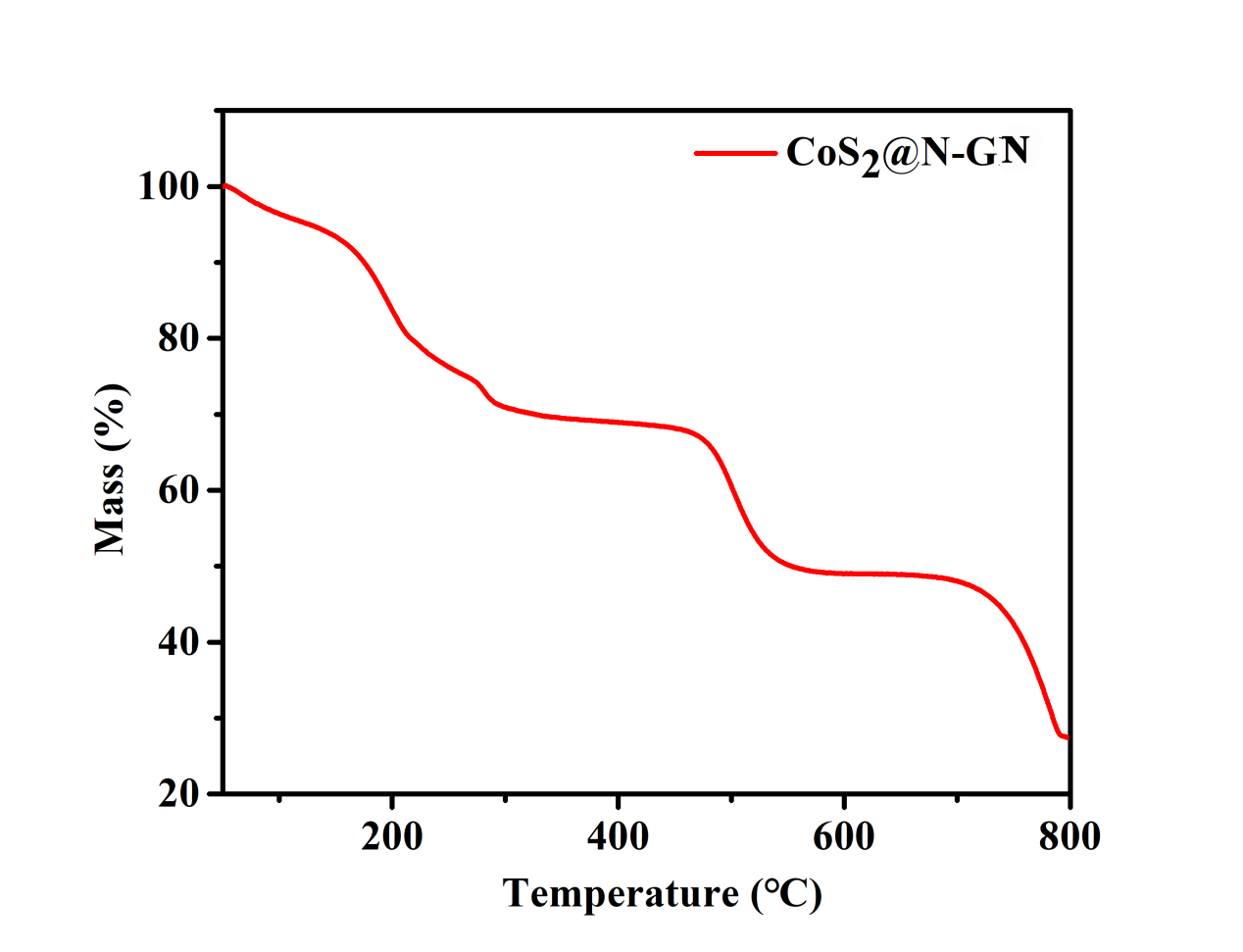


**Figure S3.** TGA curve of CoS_2_@N-GN hybrid.


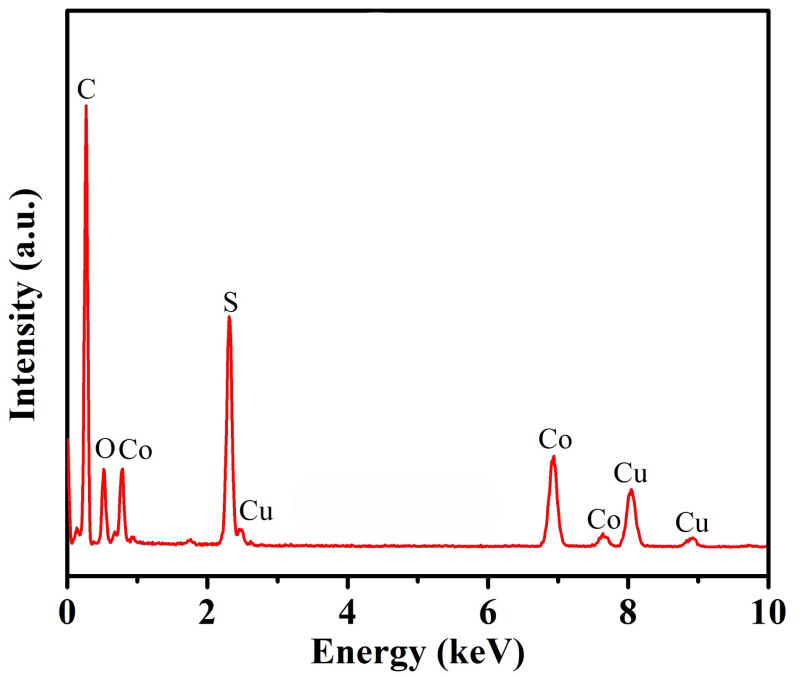


**Figure S4.** EDS of CoS_2_@N-GN hybrid.





**Figure S5.** TEM image of CoS_2_@N-GN hybrid after 500 OER cycles.


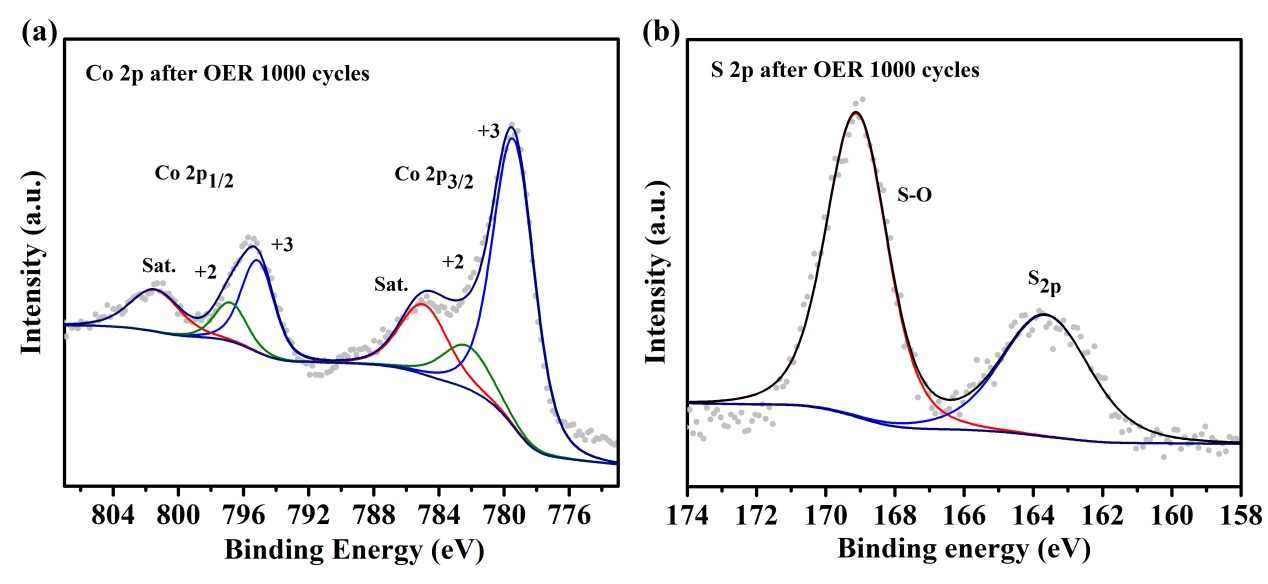


**Figure S6.** XPS spectraof the CoS_2_@N-GN hybrid after OER for 1000 cycles.


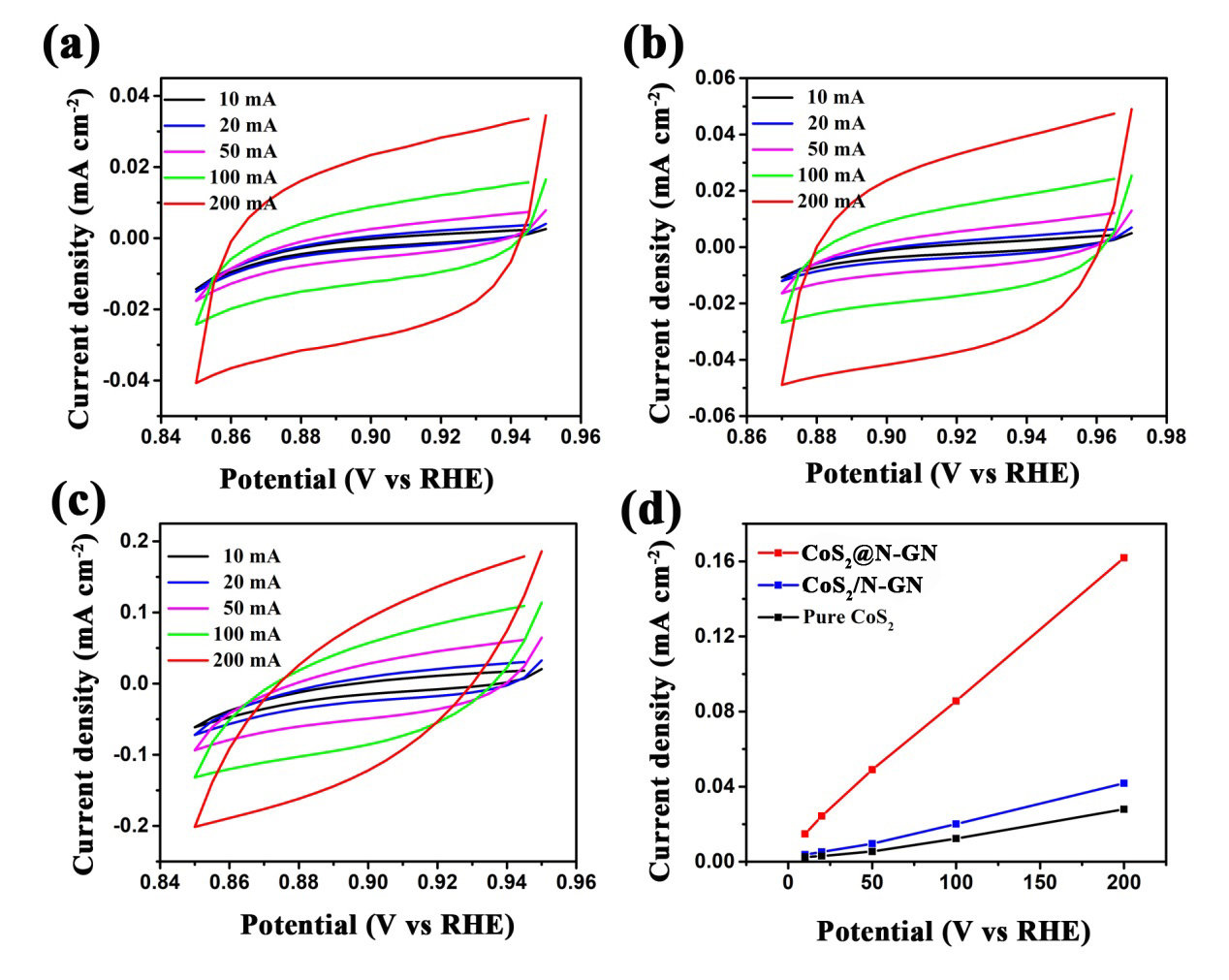


**Figure S7.** (a-c) The CVs collected at different scanning rates of pure CoS_2_ (a), CoS_2_/N-GN (b), and CoS_2_@N-GN (c), respectively. (d) The curves of the current density vs. the scanning rate of the three samples. The CVs displayed an approximate rectangular shape, indicating the current is solely from the contribution of electrical double layer discharging/charging. The profile of the current density against the scanning rate exhibits a linear relationship and the double layer capacitance (C_dl_) values can be calculated from the slopes.





**Figure S8.** TEM image of CoS_2_@N-GN hybrid after HER for 500 cycles.

**Table S1.** Comparison of OER performances of CoS_2_@N-GN with other reported similar non-noble metal OER electrocatalysts.

| Catalyst | Electrode | Electrolyte | Loading density  (mg cm^-2^) | Current density  (mA cm^-2^) | Overpotential  （mV） | Refs |
| --- | --- | --- | --- | --- | --- | --- |
| Co-P film | copper foil | 1.0M KOH | 2.71 | 10 | 345 | Energy Environ. Sci. 2013, 6, 2921-2924 |
| Zn-Co-S NN | Carbon fiber paper | 1.0M KOH | 0.6 | 10 | 320 | ACS Appl. Mater. Interfaces 2017, 9, 12574−12583 |
| NiCo_2_S_4_@N/S-rGO | glassy carbon electrode | 0.1 M KOH | 0.283 | 10 | 470 | ACS Nano 2014, 8, 3970-3978 |
| Ni3S2/Ni | Ni foam | 1.0 M KOH | 37.00 | 10 | 187 | Energy Environ. Sci. 2013, 6, 2921-2924. |
| NiP nanoparticle film | copper foam | 1.0 M KOH | 5 | 10 | 325 | Angew. Chem., Int. Ed. 2014, 53, 4372-4376 |
| Co_3_O_4_ NCs | Carbon fiber paper | 1.0 M KOH | 0.35 | 20 | 360 | Adv. Mater. 2015, 27,  3175-3180 |
| NiCo_2_O_4_nanoneedles | FTO | 1.0 M KOH | 0.53 | 20 | 807 | Angew. Chem., Int. Ed. 2012, 51, 12703-12706 |
| Ni_0.33_Co0_.67_S  nanowires | Ti foam | 1.0 M KOH | 0.30 | 10 | 330 | J. Power Sources 2014, 266, 365-373 |
| NG-CoSe_2_ | glassy carbon electrode | 0.1 M KOH | 0.20 | 10 | 366 | Energy Environ. Sci. 2014, 7, 2624-2629 |
| CoS_2_@N-GN | Carbon fiber cloth | 1.0M KOH | 1.5 | 10 | 243 | This work |

**Table S2.** Comparison of HER performances of CoS_2_@N-GN with other reported similar non-noble metal HER electrocatalysts.

| Catalyst | Electrode | Electrolyte | Loading density  (mg cm^-2^) | Current density  (mA cm^-2^) | Overpotential  （mV） | Refs |
| --- | --- | --- | --- | --- | --- | --- |
| CoOx/CN | Carbon nanotube | 1.0M KOH | 0.42 | 10 | 260 | J. Am. Chem. Soc. 2015, 137, 2688-2694 |
| Zn-Co-S NN | Carbon fiber paper | 1.0M KOH | 0.6 | 10 | 2 | ACS Appl. Mater. Interfaces 2017, 9, 12574−12583 |
| Co-NRCNTs | Carbon nanotube | 1 M KOH | 0.28 | 10 | 370 | Angew. Chem., Int. Ed. 2014, 53,4372-4376 |
| PCPTF | glass microscope slides | 1.0 M KOH | 0.1 | 10 | 807 | Adv. Mater. 2015, 27, 3175-3180 |
| FeP NAs | Carbon cloth | 1.0 M KOH | 1.5 | 20 | 370 | ACS Catal.  2014, 4, 4065-4069 |
| MoB | carbon paste electrode | 1.0 M KOH | 2.3 | 20 | 210 | Angew. Chem., Int. Ed. 2012, 51, 12703-12706 |
| MoP | glassy carbon electrode | 1.0 M KOH | 0.86 | 10 | 150 | Energy Environ. Sci. 2014, 7, 2624-2629 |
| Co0.6Mo1.4N2 | glassy carbon electrode | 0.1 M HClO4 | 0.24 | 10 | 200 | J. Am. Chem. Soc. 2013, 135, 19186-19192 |
| CoS_2_@N-GN | Carbon fiber cloth | 1.0M KOH | 1.5 | 10 | 243 | This work |
